# Brain, behavior, and physiological changes associated with predator stress–An animal model for trauma exposure in adult and neonatal rats

**DOI:** 10.3389/fnmol.2024.1322273

**Published:** 2024-02-29

**Authors:** Stacey L. Kigar, Amelia Cuarenta, Carla L. Zuniga, Liza Chang, Anthony P. Auger, Vaishali P. Bakshi

**Affiliations:** ^1^Department of Psychiatry, University of Cambridge, Cambridge, United Kingdom; ^2^Department of Medicine, University of Cambridge, Cambridge, United Kingdom; ^3^Neuroscience Institute, Georgia State University, Atlanta, GA, United States; ^4^Department of Psychiatry, University of Wisconsin School of Medicine and Public Health, Madison, WI, United States; ^5^College of Agricultural and Life Sciences Academic Affairs, University of Wisconsin-Madison, Madison, WI, United States; ^6^Department of Psychology, University of Wisconsin-Madison, Madison, WI, United States

**Keywords:** epigenetics, amygdala, post-traumatic stress disorder, biological sex, ferret, development, predator

## Abstract

The use of predators and predator odor as stressors is an important and ecologically relevant model for studying the impact of behavioral responses to threat. Here we summarize neural substrates and behavioral changes in rats resulting from predator exposure. We briefly define the impact predator exposure has on neural targets throughout development (neonatal, juvenile, and adulthood). These findings allow us to conceptualize the impact of predator exposure in the brain, which in turn may have broader implications for human disorders such as PTSD. Importantly, inclusion of sex as a biological variable yields distinct results that may indicate neural substrates impacted by predator exposure differ based on sex.

## Introduction

Live predator exposure as studied by the Blanchard et al. (1990) has remained an important method for examining stress-induced alterations in animals for several decades. The fundamental premise is that encounters with a predator recruit hard-wired fear circuitry in prey species, leading to a series of species-specific defensive behaviors that evolved to ensure an animal’s survival in the face of predatory threat. One hypothesis is that various forms of psychopathology in humans may represent either an exaggerated fear response to a threatening stimulus, or a “regular” fear response to an objectively non-threatening situation (Bakshi et al., 2000). Hence, predator stress represents an important tool for examining both maladaptive and adaptive responses to threat in preclinical models. Here, we summarize our group’s work with predator exposure in rats, describing the neural substrates and behavioral sequelae of such exposure across various developmental stages. We furthermore identify mechanisms that could contribute to either enhanced susceptibility to this unique type of stress exposure (“risk”) vs. those that could play a protective role (“resilience”) in the progression toward psychopathology-like endophenotypes.

## Predator stress exposure in adults

**FIGURE 1:**
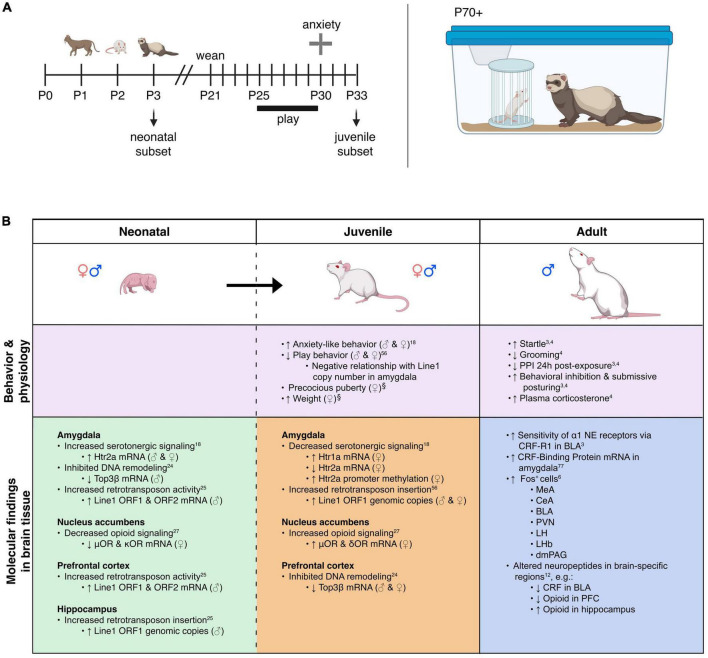
Model overview and summary of our groups’ findings on predator exposure effects across multiple developmental stages in Sprague-Dawley rats. **(A)** *Left:* Timeline for predator odor exposure at neonatal stage (see Kigar et al., 2017 for more detail). *Right:* Adult studies consisted primarily of live predator exposure (see Jochman et al., 2005; Bakshi et al., 2012; Rajbhandari et al., 2015 for more detail). All studies were done in adult male rats, purchased from Harlan Labs, aged P70 or older, and commercially available ferrets who were maintained on an *ad libitum* food and water schedule. Further methodological details can be found in the references that are cited in the text. **(B)** Summary of published behavioral, physiological, and molecular effects observed using predator stress models. All results are presented as change relative to control animals. ♀ and An error in the conversion from LaTeX to XML has occurred here. status assigned based on physical assessment of genitalia. BLA, basolateral amygdala; CeA, central amygdala; CRF, corticotropin-releasing factor; CRF-R1, corticotropin-releasing factor receptor 1; dmPAG, dorsomedial periaqueductal gray; LH, lateral hypothalamus; LHb, lateral habenula; MeA, medial amygdala; NE, noradrenergic; OR, opioid receptor; ORF, open reading frame; P, postnatal day; PFC, prefrontal cortex; PPI, pre-pulse inhibition; PVN, paraventricular nucleus of the hypothalamus; top, topoisomerases. ^§^Data presented in Figure 2.

We developed a model for trauma-like stress based on exposure (of rats) to a live predator (ferrets). Briefly, a rat is placed within a small metal protective cage that is then secured inside of a ferret’s home cage for 5 min (Figure 1A). The ferret is allowed to freely explore the small cage containing the rat; it is not unusual for the ferret to maintain constant contact with the small cage by sniffing, licking, and biting it as well as batting at it with its paws, presumably in an attempt to gain access to the rat contained within it (Jochman et al., 2005; Bakshi et al., 2012; Rajbhandari et al., 2015). This trauma-like paradigm is ethologically relevant (ferrets are predators of rats), naturalistic (encounters between rats and ferrets would occur in the wild), purely psychological (the species are never in physical contact but can see, hear, and smell each other), and intense (rats freeze then quickly go into submissive posture with continuous vocalizations and concomitant high-level corticosterone release) (Blanchard et al., 1990; Jochman et al., 2005; Bakshi et al., 2012; Rajbhandari et al., 2015). Hence, this represents a powerful method to study innate threat/fear mechanisms that produce acute and long-term effects that are distinct from those of standard laboratory-based stress paradigms (Jochman et al., 2005; Bakshi et al., 2012; Rajbhandari et al., 2015). The trauma-like element is comprised of the rat not knowing the ferret cannot enter the protective cage and likely considering the encounter life-threatening; the rat’s submissive posture and vocalizations during ferret exposure support this notion.

Our previous work shows that ferret exposure elicits non-habituating hypothalamic-pituitary-adrenal (HPA)-axis activation and behavioral effects plus unique brain activation profiles compared to standard lab stressors such as foot shock (Baisley et al., 2011). As discussed previously (Baisley et al., 2011), live predator exposure also promotes more widespread / intense activation stress-related brain regions than odor-only exposure. Furthermore, our model indicates that live ferret exposure triggers a cascade of events in the amygdala ultimately resulting in long-lasting sensitization. In turn, this long-term effect causes the emergence of a PTSD-like phenotype that captures several of the behavioral, neuroanatomical, and neurochemical factors that are widely considered core to its etiology (Grillon et al., 1996; Raskind et al., 2002; Shin et al., 2006; Rajbhandari et al., 2015; Hendrickson and Raskind, 2016). Specifically, we found that ferret exposure results in long-lasting sensitization of basolateral amygdala (BLA) pyramidal neurons such that even a month after the exposure has ended, predator-exposed rats show exaggerated defensive behaviors [startle hyperreactivity and deficits in pre-pulse inhibition (PPI), a pre-attentional form of information-processing] as seen in PTSD (Grillon et al., 1996; Rajbhandari et al., 2015)]. Following repeated predator stress these behavioral effects are elicited by low-level noradrenergic (NE) receptor stimulation specifically within the BLA. Moreover, we have demonstrated that the cellular substrate for this phenomenon is a corticotropin-releasing factor1 (CRF1) receptor-dependent sensitization of α1 NE receptors that are almost completely (>96%) colocalized with the CRF1 receptor selectively on BLA pyramidal neurons, which are the main output projection neurons of the amygdala. This ferret exposure-induced neuroplasticity renders these neurons highly sensitive to subsequent stimulation with subthreshold levels of noradrenergic transmission (Rajbhandari et al., 2015). Hence, we have shown that predator stress can reset the sensitivity of the output projection pathways of the BLA. Given the central role of the BLA to numerous processes including cognition, learning, memory, perception, and affective processing, these findings suggest that enhanced NE receptor sensitivity resulting from CRF1 receptor stimulation could represent a vulnerability (“risk”) substrate after trauma-like stress exposure. We have noted that there are clear individual differences in rats exposed to the ferret stress paradigm (unpublished results), with some showing super-sensitization of BLA NE α1 receptors and others showing no such effect. Hence, rats that fail to develop NE receptor sensitization in the aftermath of predator stress may represent a “resilient” subset (approximately 10% of the cohort). The cellular and molecular substrates that either drive or prevent this post-trauma sensitization from developing remain to be determined in future studies. A recent study from our group illustrating unique peptidomic signatures of predator stress in the BLA suggests several potential candidates including members of the tachykinin family such as Substance P (Wu et al., 2023). Our findings are summarized in Figure 1B.

Predator exposure results in marked individual differences in behavioral responses; extreme behavioral inhibition (enhanced freezing and hypervigilance) is seen in a subset of rats and this is a stable trait-like feature that is positively correlated with expression of the plasticity-related gene Homer1a in the hippocampus and paraventricular nucleus of the hypothalamus (Qi et al., 2010). We have found that live predator exposure elicits neuronal activation (assessed by Fos protein expression) at much higher levels in the medial amygdala compared to standard lab stressors (Baisley et al., 2011). The function of this neuroanatomical site should be investigated further as a putative substrate for susceptibility to trauma-like exposure, or alternatively resilience to trauma (i.e., via recruitment to counteract negative effects of trauma exposure). Individual differences in response to live predator exposure could to some extent also reflect changes in neuropeptide signaling. Recently, we characterized peptidomic profiles in multiple trauma-related brain regions in predator exposed rats compared to controls. Clear stress- and site-specific differences were seen in a variety of signaling peptides, with the biggest effects seen in the basolateral amygdala (Wu et al., 2023). These findings are generally consistent with work from Cohen and colleagues showing that neuropeptide Y levels in the amygdala were inversely correlated with the intensity of defensive behaviors after predator stress (Cohen et al., 2012). Thus, neuropeptide Y could also contribute to stress “resilience” in response to predator exposure. Ongoing studies examining individual differences in these responses will facilitate identification of molecules conferring risk or resilience following trauma-like exposure.

One key factor that determines the effects of predator exposure (in rats) or trauma exposure (in humans) is the developmental stage at which that exposure occurs. For example, we have seen that live predator exposure in adolescent rats produces a much larger neuroendocrine response than it does in adult rats (unpublished data). Nevertheless, the largest body of literature, both in rats and in humans, concerns stress exposure in early life. Observational studies suggest approximately 45% of childhood-onset and 30% of adult-onset mental health disorders are associated with prior early life stress (ELS) exposure (Green et al., 2010). There is robust literature on ELS and the long-lasting sequelae of those experiences, both in rodents and in humans (White and Kaffman, 2019; Hanson et al., 2021). It is beyond the scope of the present article to summarize that entire body of work. Here, we focus specifically on predator exposure during the early postnatal period in rats to illustrate the unique effects of this ethologically relevant, trauma-like stress at different developmental stages. The unconditioned nature of predator stress makes it suitable for use in neonatal rodents which otherwise lack adult-like fear learning until at least the second week of life (Moriceau et al., 2010).

## Predator odor exposure (POE) stress in neonates

We adapted our live ferret exposure model for use with neonatal rats by placing litters of pups on an elevated platform in an enclosure containing soiled bedding from natural predators (cats, unfamiliar adult male rats, or ferrets); no direct contact with the odor occurred (Kigar et al., 2017) (Figure 1A). The exposure lasted 5 min and occurred on 3 consecutive days (P1–P3, with the day of birth defined as postnatal day 0); different predator odors were used each day to avoid potential habituation (Staples, 2010). The period between P1 and P3 is developmentally distinct given that epigenetic and cellular reorganization of the brain by estrogen and testosterone is occurring during this time (Kigar and Auger, 2013; McCarthy et al., 2015).

Within 30 min of the last predator exposure at P3, we find in the amygdala increased expression of the Htr2a serotonin receptor in males and females (Kigar et al., 2017). Consistently, other labs have reported increased neuronal activation in the amygdala within 2 h of predator odor exposure (POE) cessation (Wiedenmayer and Barr, 2001a; Moriceau et al., 2004). In males (but not females) we find decreases in DNA-remodeling enzymes related to genomic integrity (Cuarenta et al., 2023) and increases in retrotransposon translation and insertion, resulting in increased gene copy number (Cuarenta et al., 2020)—the latter being an interesting contrast to work showing suppression of retrotransposon activity by maternal care (Bedrosian et al., 2018). These effects were present in multiple brain regions, including the PFC, amygdala, and hippocampus. In females (but not males) we found decreased expression of μ- and κ-opioid receptors in the nucleus accumbens (NAc) (Chang et al., 2019). It is not presently clear what functional or developmental role these receptors play at P3, but other groups studying predator odor-induced analgesia in older neonates have also demonstrated changes in opioid receptor signaling pathways (Wiedenmayer and Barr, 1998, 2000, 2001b; Wiedenmayer et al., 2002); future research in this area is needed. For a summary of our work, see Figure 1B. Other labs have reported acute effects of predator stress in older neonates, including reduced emission of ultrasonic vocalizations [USVs, (Conely and Bell, 1978; Lyons and Banks, 1982; Lemasson et al., 2005)], increased freezing (Bronstein and Hirsch, 1976; Gould and Cameron, 1997; Wiedenmayer and Barr, 1998, 2000, 2001a,2001b; Wiedenmayer et al., 2003a,^2005^; Moriceau et al., 2004), or both (Takahashi, 1992b,1994a,1994b,^1995^; Takahashi and Rubin, 1993; Takahashi and Kim, 1994, 1995; Shair et al., 1997; Wiedenmayer et al., 2003a; Ayers et al., 2016; Stockman and McCarthy, 2017). While freezing is an adult-like defensive behavior, suppression of USVs may be more specific to neonates and used to evade detection by an infanticidal adult male or other predator (Takahashi, 1992a). Involvement of the hippocampus in mediating these effects has been demonstrated by several groups (Takahashi, 1995; Gould and Cameron, 1997; Stockman and McCarthy, 2017). Several labs have reported increased plasma corticosterone within 45 min of POE stress cessation (Tanapat et al., 1998; Wiedenmayer et al., 2005; Stockman and McCarthy, 2017) and decreased hippocampal neurogenesis 24 h later (Tanapat et al., 1998; Stockman and McCarthy, 2017).

### Epigenetic reorganization following neonatal predator exposure, and long-term effects

**FIGURE 2:**
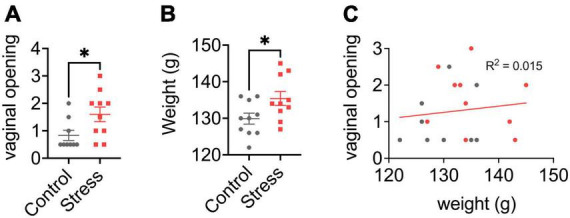
Effects of neonatal predator exposure on **(A)** vaginal opening and **(B)** body weight in adolescent females (at postnatal day 33). **(C)** Lack of correlation between vaginal opening and body weight (*R*^2^ = 0.02, not significant). Vaginal opening was assessed on a scale from 0 (completely closed) to 3 (completely open) by an experienced observer blind to condition. These data were collected from an animal cohort described in previous publications. Please see ref. Kigar et al. (2017) for more detail. Values represent means ± s.e.m. *p < 0.05.

Very few non-adult POE studies have investigated long-term effects of the stress exposure. Our paradigm was designed to test the hypothesis that disruptions caused by predator exposure during the “sensitive window” of brain sexual differentiation would have lasting effects on juvenile behavior, given extensive work from our lab on epigenetic reorganization occurring during this period (Olesen et al., 2005; Kurian et al., 2008; Jessen et al., 2010; Edelmann et al., 2013; Kigar et al., 2015). Profound long-term behavioral effects of neonatal POE were seen in adolescence (30 days after the predator exposure): decreased juvenile social play (Cuarenta et al., 2021), disrupted PPI (data unpublished), and increased anxiety-like behavior (Kigar et al., 2017). To our knowledge we are the only lab to have examined long-term molecular changes in the juvenile brain occurring due to neonatal POE (see Figure 1B). Briefly, we find female-specific changes indicative of decreased serotonergic signaling in the amygdala (i.e., increased expression of the Htr1a autoreceptor, decreased expression of Htr2a) that are associated with increased DNA methylation (Kigar et al., 2017). Females also expressed higher levels of μ- and κ-opioid receptors in the NAc, but not the amygdala (Chang et al., 2019). Expression of opioid receptors in the NAc is thought to be involved with hedonic perception of food (Baldo and Kelley, 2007), which may be consistent with increased weight gain observed in these animals (Figure 2B). Both males and females show increased insertion of Line1 retrotransposon into the genome; this insertion negatively predicts levels of juvenile social play (Cuarenta et al., 2021). Further, males and females exhibit decreased expression of DNA topoisomerase Top3β in the PFC, perhaps indicating decreased resiliency to further genomic insults (Cuarenta et al., 2023). Collectively this work suggests that neonatal exposure to POE, even at a developmental time point when defensive behaviors may not yet be evident, causes lasting effects on a variety of behaviors via reorganization of the genome at several levels, including DNA (Kigar et al., 2017; Cuarenta et al., 2020, 2021, 2023), RNA (Kigar et al., 2017; Chang et al., 2019; Cuarenta et al., 2020, 2021, 2023), chromatin (Kigar et al., 2017), and retrotransposon expression (Cuarenta et al., 2020, 2021).

### Consideration of biological sex

Relatively few POE studies, or preclinical studies of different stressors in general, explicitly examine biological sex in their analyses. We have shown in male and female rats that POE at a very young age leads to decreased juvenile social play behavior from P25 to P30 (Cuarenta et al., 2021) and increased anxiety-like behavior in the elevated plus maze (EPM) at P30 (Kigar et al., 2017). Despite similar behavioral results in juvenile males and females, we find many sex-, age-, and regionally specific molecular effects of neonatal predator exposure (Figure 1B). A similar study reported elevated corticosterone 45 min after the last exposure to stress in males only (Stockman and McCarthy, 2017). However, the Stockman and McCarthy study did not replicate increased juvenile anxiety-like behavior and decreased play behavior in females reported by our lab; while there are small technical differences in experimental design and data analysis between the two studies, we cannot presently account for this discrepancy and future efforts to reconcile the data are warranted. Interestingly, Stockman and McCarthy report POE exposure in early life has a lasting effect on adult anxiety-like behavior in the open field test; given our preliminary data concerning early puberty initiation in POE-stressed animals (see below) it is possible that day of testing will be an important consideration. Similarly, estrous cycle stage (not measured in our studies) could modulate stress effects as well. Collectively, these data suggest males and females may arrive at similar behavioral outcomes by different mechanisms. It would be reasonable to hypothesize that genetic risk factors or secondary environmental insults affecting a predominately “male”- or “female”- specific pathway would manifest differently depending on the biological sex of the individual. This type of sex-specific risk and/or resilience has been discussed elsewhere (Kigar and Auger, 2013; Bangasser and Cuarenta, 2021), and underscores the importance of considering males and females separately, even when behavioral output is similar, as sex-specific therapeutic strategies may yield better treatment outcomes.

### Precocious puberty in females

We report for the first time here that neonatal POE exposure leads to early vaginal opening in juvenile females (Figure 2A). Mixed effects on puberty timing have been shown in other models of early life stress (Lau et al., 1996; Cowan and Richardson, 2019; Manzano Nieves et al., 2019; Davis et al., 2020), but to our knowledge this is the first report of such an effect with early life exposure to predator odor. Investigation of pubertal timing was not a planned comparison in our POE study, and we did not explore altered puberty in males. However, other models of ELS have found divergent effects on males and females; specifically, early puberty in females and delayed puberty in males (Cowan and Richardson, 2019; Davis et al., 2020), but see also (Lau et al., 1996; Manzano Nieves et al., 2019). In humans, early puberty in females—clinically defined by early menarche, thelarche, and pubarche—is associated with deleterious outcomes such as increased risk for breast (Collaborative Group on Hormonal Factors in Breast Cancer, 2012) and endometrial (Gong et al., 2015) cancer. This highlights an important, translationally relevant aspect of our neonatal POE model given that in girls severe childhood trauma (Noll et al., 2017) and stress reactivity (Colich et al., 2015) are associated with precocious puberty. Severe childhood trauma is also associated with higher BMI (Trickett et al., 2011; Danese and Tan, 2014)—itself a well-known risk factor for precocious puberty (Biro et al., 2013; Brix et al., 2020). Interestingly, in our POE-exposed females we observed a concomitant increase in weight at P33 (Figure 2B) that was not associated with vaginal opening score (Figure 2C). This suggests that POE stress in early development may provide a useful model for disentangling the effects of stress on obesogenic and precocious pubertal outcomes. Future efforts extending this model into males are also needed.

## Discussion

Predator exposure, both in adulthood and in the immediate postnatal period, clearly causes multiple stress-related acute and chronic effects that are distinct from those of other standard lab stressors. This likely reflects the fact that predator exposure taps into hardwired, unlearned, innate neural substrates that evolved to ensure survival of an individual in the face of life-threatening danger. As such, predator exposure offers an ethologically valid model for examining trauma-like stress in rodents with translational utility in studying substrates of PTSD (Zoladz and Diamond, 2016). Individual differences in responsivity to predator exposure may thus offer clues about brain mechanisms underlying susceptibility vs. resilience to trauma.

In particular, we have shown that predator stress-induced neuroplasticity of NE receptors within the basolateral amygdala governs behavioral risk or resilience following predator exposure in adult rats. In neonates, predator odor exposure causes differences in genomic regulation and neurotransmitter signaling pathways, but intervention-based studies are still needed. One potential intervention strategy could be increased maternal care. Two groups have reported effects of daily pup exposure to predator odor in the presence of the dam for the first 3 weeks of life. Surprisingly, exposure did not cause behavioral inhibition in the dam, but elevated maternal behaviors (Ayers et al., 2016). Naturally occurring variations in maternal care during early development influence stress reactivity later in life (Weaver et al., 2004); higher engagement in maternal behavior would thus be expected to reduce stress and anxiety-like behaviors. Indeed, re-exposure to predator odor, either during adolescence (Ayers et al., 2016) or adulthood (Hacquemand et al., 2010) blunted avoidance behavior compared to control litters; acutely, reunion with the dam after a single POE significantly decreases plasma corticosterone (Wiedenmayer et al., 2003b). Further, non-cycling adult females from the odor-exposed litters were less anxious (Hacquemand et al., 2010) and performed better in tests for working and spatial memory (Hacquemand et al., 2012). It is interesting to consider whether this is a positive or negative outcome; anthropomorphically, one might assume better memory and less anxiety are desirable, but ethologically, less behavioral inhibition in this context may increase susceptibility to predation. Nevertheless, these data clearly indicate the importance of the early social environment in mitigating a behavioral response to fearful stimuli. Interestingly, distinct groups of susceptible and resilient animals may emerge at P21; an all-male study found approximately half of the animals showed freezing behaviors with concomitantly distinct patterns of neuronal activation compared to the remaining POE animals (Wiedenmayer and Barr, 2001a). Future efforts to characterize factors leading to resilience and susceptibility in the neonatal period will be important. Together, these data indicate that psychological stressors associated with fear of harm as modeled by predator exposure have lasting consequences on brain, behavior, and physiology. These consequences appear to mimic PTSD-like symptoms, which can occur when a traumatic stressor exceeds allostasis (McEwen, 2000), resulting in lasting pathological changes.

## Data availability statement

The original contributions presented in the study are included in the article/supplementary material, further inquiries can be directed to the corresponding author.

## Ethics statement

The animal study was approved by the University of Wisconsin-Madison IACUC. The study was conducted in accordance with the local legislation and institutional requirements.

## Author contributions

SK: Conceptualization, Writing – original draft, Writing – review and editing, Data curation. AC: Writing – review and editing. CZ: Writing – review and editing. LC: Writing – review and editing. AA: Conceptualization, Writing – review and editing. VB: Conceptualization, Supervision, Writing – original draft, Writing – review and editing.
